# The influence of Wi-Fi on the mesonephros in the 9-day-old chicken embryo

**DOI:** 10.1007/s11259-025-10777-x

**Published:** 2025-06-10

**Authors:** Viera Almášiová, Sandra Andrašková, Viera Karaffová, Patrícia Hudáková, Ján Molnár, Štefan Tóth, Katarína Holovská

**Affiliations:** 1 Department of Morphological Disciplines, University of Veterinary Medicine and Pharmacy in Košice, Komenského 73, Košice, Slovakia; 2 Department of Theoretical and Industrial Electrical Engineering, Faculty of Electrical Engineering and Informatics, Technical University of Košice, Letná 9, Košice, Slovakia; 3 Department of Histology and Embryology, Faculty of Medicine, Pavol Jozef Šafárik University in Košice, Šrobárova 2, Košice, Slovakia

**Keywords:** Wi-Fi, Chicken embryo, Mesonephros, Development

## Abstract

The use of wireless devices has increased rapidly in recent times, especially in developed countries. As a result, all living systems are to some extent permanently exposed to this artificial electromagnetic non-ionizing radiation (NIR). These modern devices provide countless benefits to the users, but the disadvantage of their excessive use is the production of electrosmog. This physical pollutant of the environment can be particularly dangerous especially during the developmental period of the individual. The aim of the current study was to elucidate the effect of Wi-Fi radiation on the mesonephros development in the chicken embryo on day 9 of incubation. Continual 9-day application of radiation with a frequency of 2.4 GHz and a power density of 200—500 µW/m^2^ had no adverse effect on the general development of the mesonephros, however moderate diffuse degenerative changes were found in the developing mesonephric corpuscles and tubules. Also congested blood vessels were present in the surrounding interstitium, but no signs of inflammatory infiltrate were detected. In the Wi-Fi group, we also noted a significantly increased number of apoptotic and proliferating cells as well as a significant up-regulation of caspase-1 gene expression. The results indicated that non-ionizing radiation at the frequency and power density used in the study can interfere with the key regulatory mechanisms involved in the normal development of tissues and organs.

## Introduction

Wi-Fi has become a daily routine of modern society, as it allows very convenient connection to the internet and various data in different places. Public areas with an easily achievable internet connection, the so called hotspots are spread almost over everywhere. Due to such a considerable expansion of the non-ionizing radiation (NIR) produced by modern devices such as Wi-Fi, mobile phones, base stations and other wireless technologies used by the human population on a daily basis, one can presume its massive and cumulative nature. Wireless technologies usually work on the basis of pulsations, which are more dangerous to the body than the continuous propagation of electromagnetic waves (Van Boxem et al. 2014; Pall 2018). These man-made waves are also polarized and therefore more dangerous for the living matter than the non-polarized waves (Panagopoulos et al. 2015). The exposure windows, where a significant biological response occurs at the specific intensity have also been revealed (Foletti, et al. 2010; Pall 2018). Overall, even if the body's exposure to artificial NIR in the common environment is many times weaker than the exposure limits set by the International Commission on Non-Ionizing Radiation Protection—ICNIRP (ICNIRP 2020) as well as IEEE Std C95.1–2019 (IEEE 2019) it will be necessary to study this issue in detail, especially if it concerns susceptible individuals such as young and developing organisms (Redmayne and Johansson 2015; Xu et al. 2016; Sage and Burgio 2017; Woelders et al. 2017; Othman et al. 2018).

The chick embryo is an ideal model organism to study the effects of various environmental pollutants on embryonic development due to its undemanding availability, convenient handling and easy observation of early stages of embryogenesis. Since this is an oviparous species, any response to externally applied NIR can be attributed to its direct interaction with the developing organism and is not influenced by the mother (Ubeda et al. 1994). The entire pre-hatch or prenatal development is a very dynamic and complex process, involving intensive cell division, migration, differentiation and apoptosis, and therefore any external impulse can interfere with these sensitive processes and may lead to various adverse health consequences (Abdel-Rassoul et al. 2007; Behari 2010).

Since there is a constantly growing tendency to use wireless devices as well as introduce new frequencies of NIR for greater satisfaction of the users and, on the other hand, a great lack of information on the influence of Wi-Fi on the overall development of an individual, the aim of the current study was to evaluate its influence on the kidney development in the 9-day old chicken embryo from the morphological aspect.

## Materials and methods

### Experimental scheme

Forty (40) fertilized chicken eggs *(Gallus gallus domesticus)* of Lohmann Brown breed, purchased from the chicken farm Párovské Háje, Nitra, Slovak Republic, were randomly assigned to two control (CO 1, *n* = 10 and CO 2, *n* = 10) and two Wi-Fi (Wi-Fi 1, *n* = 10 and Wi-Fi 2, *n* = *10*) groups. The eggs were incubated at a temperature of 37.5 °C and air humidity of 60% in two separate automatic incubators (River systems ET49, Campodarsego, Italy).

The eggs from Wi-Fi groups (*n* = 20) were exposed to NIR at a frequency of 2.4 GHz and power density of 200—500 µW/m^2^ permanently during 9 days. The distance of eggs from the source of radiation (Mikrotik Metal 52 ac 15 s antenna) was 45 cm.

**Fig. 1:**
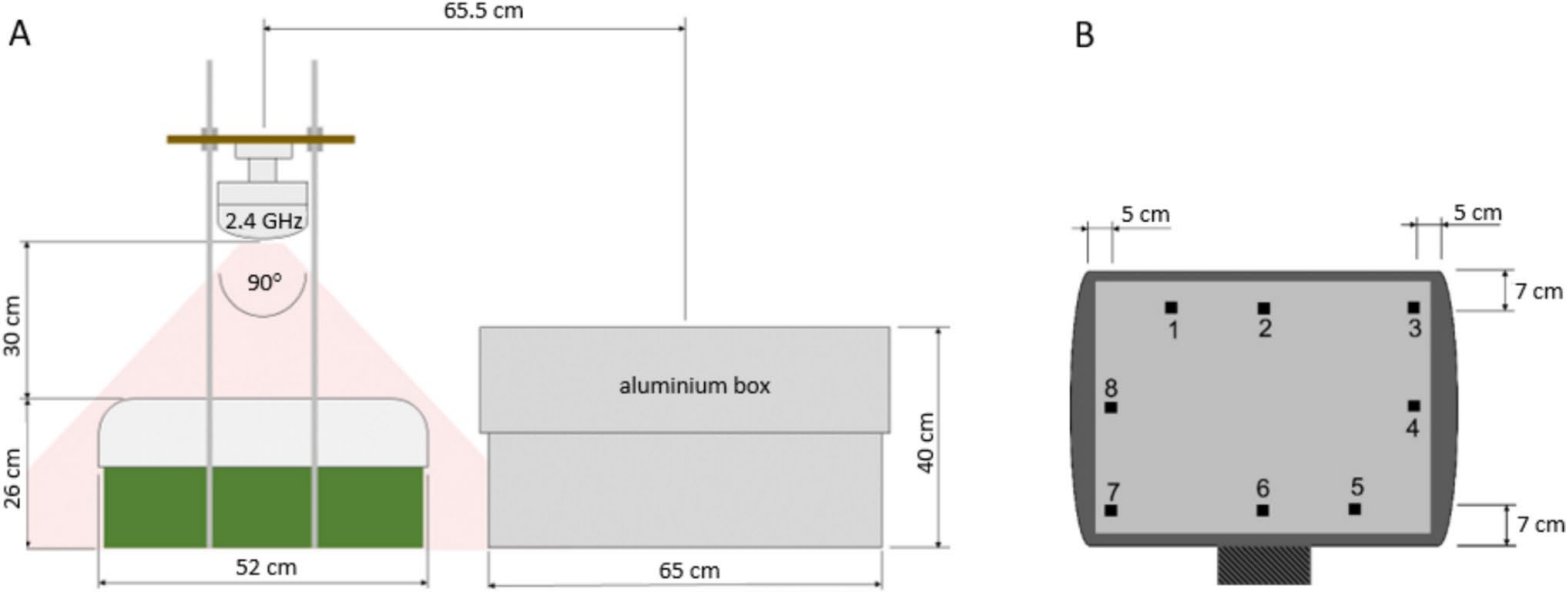
Experimental scheme. **A** – Left incubator containing eggs exposed to Wi-Fi and right incubator covered by a shielding box protecting eggs against NIR. **B** – Eight measurement points of Wi-Fi signal strength

The eggs from CO groups (*n* = 20) were incubated in an incubator shielded by an aluminium box under the same incubation conditions and the same room like the Wi-Fi groups (Fig. 1A). Before performing the experiment, evaluation of Wi-Fi signal strength at the antenna power of 10 dBm was carried out through a digital high frequency analyser for frequencies 2.4—6 GHz (HF W 35C, Gigahertz solutions GmbH, 90579 Langenzenn, Germany). Eight measurement points were selected based on the incubator's heating element and fan arrangement, above the eggs for both incubators (Fig. 1B). Results of the measurements from Wi-Fi group were further used to allocate 20 eggs into each incubator in a manner that ensures comparable Wi-Fi exposures (power density ranging from 200 to 500 µW/m^2^). The power density in the shielded incubator ranged from 1 to 7 µW/m^2^ (Table 1).

**Table 1:** Wi-Fi signal strength at different measurement points (frequency 2.4 GHz, antenna power 10 dBm, data sending 98 Mb/s)

Power density [µW/m^2^]								
Measurement points	**1**	**2**	**3**	**4**	**5**	**6**	**7**	**8**
Control group	2	1	4	6	5	3	7	2
Wi-Fi group	300	500	500	3600	300	400	200	1300

According to the Directive 2010/63/EU, the use of chicken embryos as an animal experimental model does not necessitate ethical committee approval.

### Conventional light microscopy

**Fig. 2:**
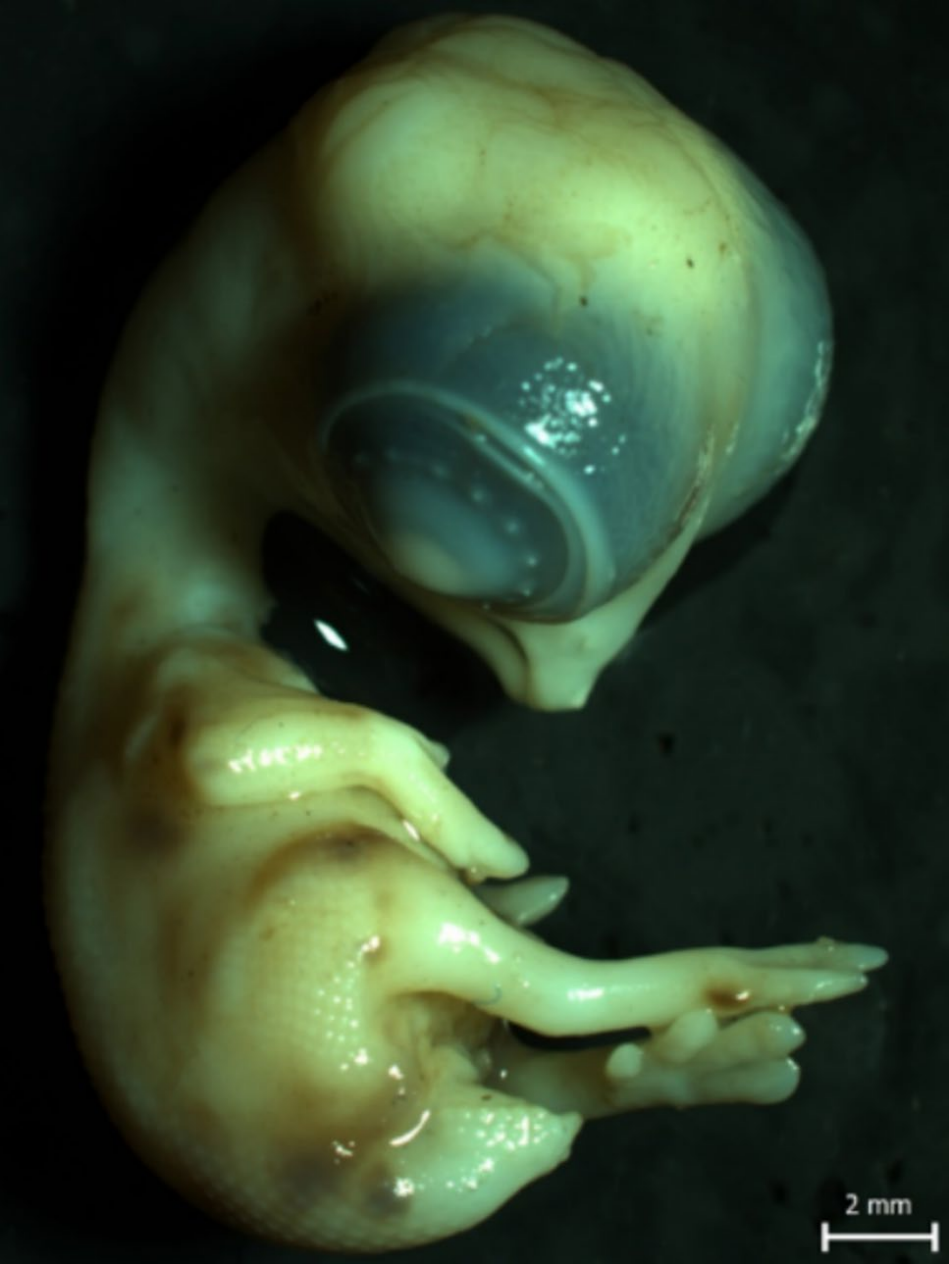
9-day-old chicken embryo (own source)

On the embryonic day 9, embryos from the CO 1 and Wi-Fi 1 group were carefully removed from the eggs, stripped of all coverings, euthanized and fixed in a modified Davidson's fluid—mDF (Latendresse et al. 2002) by conventional immersion for 24 h. Consequently, whole embryonic bodies were examined under a dissecting microscope (SZ 61) equipped with a digital Promicra camera (Fig. 2), dehydrated in a graded series of ethanol, transferred to xylene, and embedded into paraplast blocks (Sigma-Aldrich). Six (6) µm thick serial parasagittal sections through the left part of embryonic body (*n* = 10) from each block were stained with hematoxylin–eosin (HE) and evaluated with a Zeiss Axio Lab A1 light microscope and photodocumented with an Axio Cam ERc 5 camera. In each tissue section, we evaluated randomly selected fields from the caudal (10 fields), middle (10 fields) and cranial (10 fields) parts of the mesonephros.

### TUNEL detection

Four (4) µm thick serial parasagittal tissue sections from the same paraplast blocks (CO 1, *n* = 10 and Wi-Fi 1, *n* = 10), were used for the determination of apoptotic cells in the mesonephros, carried out by terminal deoxynucleotidyl transferase nick end labeling—TUNEL assay (In situ Cell Death Detection Kit, Fluorescein, Roche, Germany). Tissue sections were deparaffinised, rehydrated in graded ethanol series until water was used. Slides then were treated with 10 µg/ml proteinase K in 10 mM Tris/HCl (pH 7.5) for 30 min at room temperature. After two washes with 0.01 M phosphate buffer solution (PBS, pH 7.4) the sections were labelled with TUNEL reaction mixture in accordance with the established protocol. The labeling reaction was conducted at 37 ℃ for 60 min in a humidified chamber in dark. After washing three times in PBS, the slides were mounted using fluorescence medium (H-1200, VECTASHIELD, Vector Laboratories, Burlingame, CA, USA). Images were acquired via fluorescence microscope Nikon Eclipse H550L (Japan) connected to a digital camera (ProgRes MF JENOPTIK). They were evaluated using an excitation wavelength in the range of 450 – 500 nm and detection in the range of 515 – 565 nm (green). In each tissue section (CO 1, *n* = 10 and Wi-Fi 1, *n* = 10) ten (10) randomly selected fields (500 cells per one field) from the medial and caudal mesonephros parenchyma were evaluated at × 20 objective. Number of TUNEL positive cells was counted by two independent histologists using a Zeiss Primostar light microscope and cytometric software Quick Photo Industrial 3.2 image analysis software (Promicra). The cranial part of the mesonephros was intentionally excluded from the evaluation.

### MCM2 detection

The next 4 µm thick serial parasagital sections (*n* = 10) from the same paraplast blocks (CO 1 and Wi-Fi 1) were used to determine the proliferative activity of cells using a proliferative factor Minichromosome Maintenance Complex Component 2 (Anti-MCM2, 1:200, Abcam, UK). After deparaffinization and rehydration, endogenous peroxidase activity was blocked with 3% H_2_O_2_ in methanol. Pre-treatment was performed in a microwave oven at 600 W for 15 min in 0.01 M citrate buffer (pH 6.0). For immunohistochemical detection, all sections were treated with primary antibody overnight at 4 °C in a humidified chamber. After washing with PBS, the secondary antibody was applied to the slides for 1 h at room temperature. The positive cells were visualized with diaminobenzidine (DAB; Sigma-Aldrich, Merck, Germany), and counterstained using Mayer's haematoxylin. Similarly, in all tissue sections (CO 1, *n* = 10 and Wi-Fi 1, *n* = 10) ten (10) randomly selected fields (500 cells per one field) from the medial and caudal mesonephros parenchyma were evaluated at × 20 objective. The cranial part of the mesonephros was intentionally excluded from the evaluation as well. The number of MCM2 positive cells was counted by two independent histologists using a Zeiss Primostar light microscope and cytometric software Quick Photo Industrial 3.2 image analysis software (Promicra).

### Statistical analysis of TUNEL and MCM2 detection

The data collected from TUNEL and MCM2 detection were analysed and the statistical significance of the differences between the CO 1 and Wi-Fi 1 group was determined using a paired T-test (GraphPad Prism 8.0, USA). The data were presented as mean standard deviation (± SD). We considered the *p* < 0.05^*^ statistically significant and *p* < 0.001 ^***^ highly significant.

### Gene expression

#### Homogenization of tissue and isolation of total RNA

Embryos from the CO 2 (*n* = *10*) and Wi-Fi 2 (*n* = *10*) groups were carefully removed from the eggs, stripped of all coverings and euthanised. Under a dissecting microscope (SZ 61), the body cavity of each embryo was opened and both left- and right-sided mesonephros were collected. The samples were immediately placed in RNA Later solution (Qiagen, UK) and stored at − 70 °C prior to total RNA purification as described by Karaffová et al. (2017).

#### Quantitative real-time PCR (qRT-PCR)

Relative gene expression for caspase- 1 was measured. In addition, mRNA relative expression of reference genes, coding UB (ubiquitin) and GAPDH (glyceraldehyde-3-phosphate dehydrogenase) were selected based on obtained expression stability (geNorm software). The primer sequences and optimal annealing conditions for each primer used for qRT-PCR are listed in Table 2. All primer sets allowed DNA amplification efficiencies between 94 and 100%. Amplification and detection of specific products were performed using the Power SYBR™ Green PCR Master Mix (Thermo Scientific, USA) on a LightCycler 480 II (Roche, Switzerland). The qRT-PCR used to detect mRNA relative expression for caspase-1 was carried out over 38 cycles under the following conditions: initial denaturation at 94 °C for 3 min, subsequent denaturation at 93 °C for 45 s; annealing (Tab. 2); final extension step for 2 min at 72 °C. A melting curve from 50 °C to 95 °C with readings at every 0.5 °C was produced for each individual qRT-PCR plate. All reactions for real-time PCR were done in duplicate, and the mean values of duplicates were used for further analysis. We also confirmed that the efficiency of amplification for each selected gene (including GAPDH, UB) was essentially 100% in the exponential phase of the reaction, where the quantification cycle (Cq) was calculated. The Cq values of the studied genes were normalised to an average Cq value of the reference genes (ΔCq), and the relative expression of each gene was calculated mathematically as 2^–ΔCq^.

**Table 2:** List of primers used in qReal-Time PCR for the gene mRNA quantification

Primer	Sequence 5'–3'	Annealing/temperature time	References
Casp-1 Fw	GATACGTGACTCCATCGACCC	47 °C / 60 s	Grzegorzewska et al. 2016
Casp-1 Rev	CTTCTTCAGCATTGTAGTCC		
UB Fw	GGGATGCAGATCTTCGTGAAA	59 °C / 30 s	De Boever et al. 2008
UB Rev	CTTGCCAGCAAAGATCAACCTT		
GAPDH Fw	CCTGCATCTGCCCATTT		
GAPDH Rev	GGCACGCCATCACTATC		

### Statistical analysis of gene expression

Statistical analysis of gene expression data was done using non-parametric T- test in Graph Pad Prism version 8.00. Differences between the mean values for groups were considered statistically highly significant at *p* < *0.001.* Values are given as means with standard deviations.

## Results

### Conventional light microscopy

**Fig. 3:**
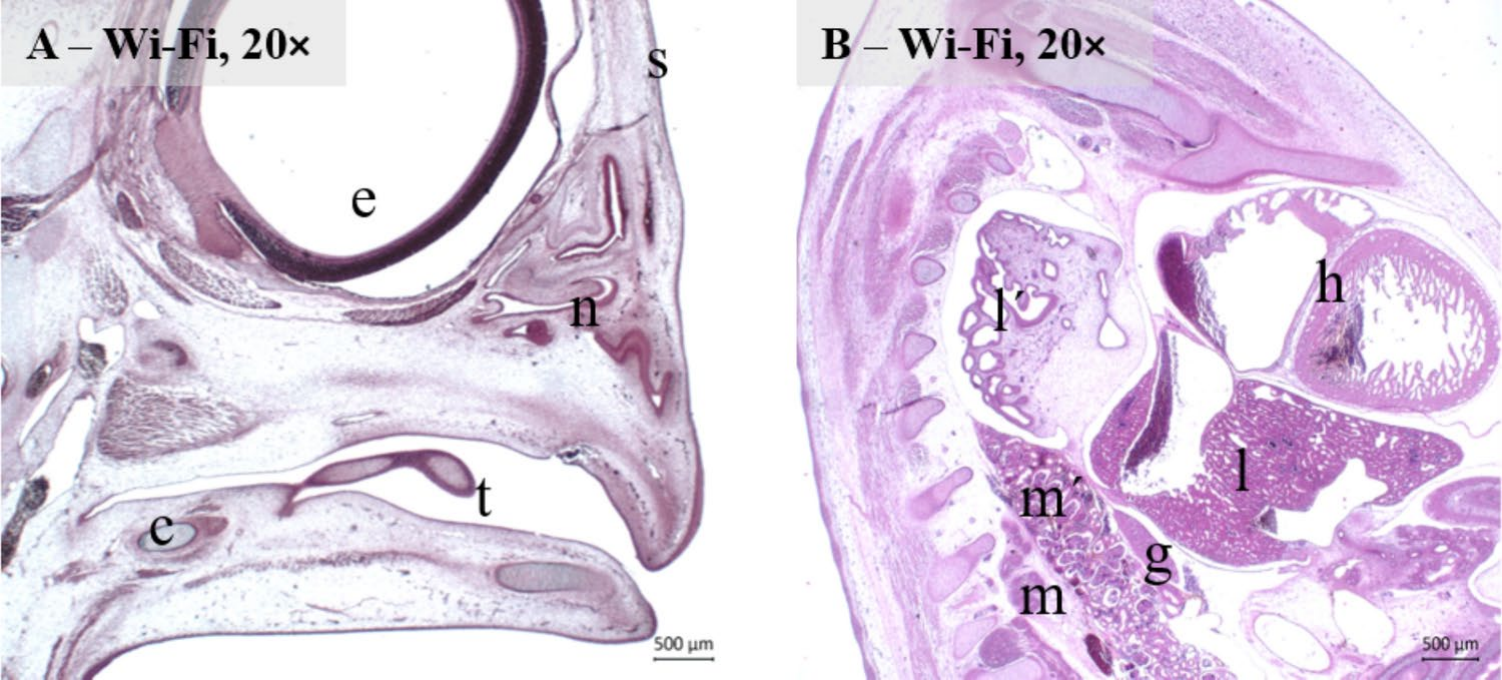
Representative microphotographs of the head **A** and body cavity **B** of the 9-day-old chicken embryo in the Wi-Fi group, s – developing skin, e – developing eye, n – developing nasal cavity, t – developing tongue, c – cartilaginous model of the mandible bone, h – developing heart, l – developing liver, l´ – developing lungs, m – developing metanephros, m´ – developing mesonephros, g – developing gonad (HE, magnification 20 ×)

In the histological sections of the chicken embryos from the CO and Wi-Fi group, we observed well-formed organs without any marked structural abnormalities. The entire body surface was covered by skin with clearly formed epidermis and adjacent mesenchyme. Within the head region, very well developed brain hemispheres, eye, nasal cavity, beak and tongue with its characteristic histological structure were found. The body cavity contained typical heart with adjacent large vessels, lungs with precursors of parabronchi, liver with developing cords of hepatocytes and spleen. Trachea, oesophagus, glandular and muscular stomach, intestines and cloaca were clearly defined too. The indifferent gonad was tightly attached to the mesonephros, in the vicinity of which the Müllerian duct was running. Small precursors of the future definitive kidney, the matanephros were also present (Fig. 3).

**Fig. 4:**
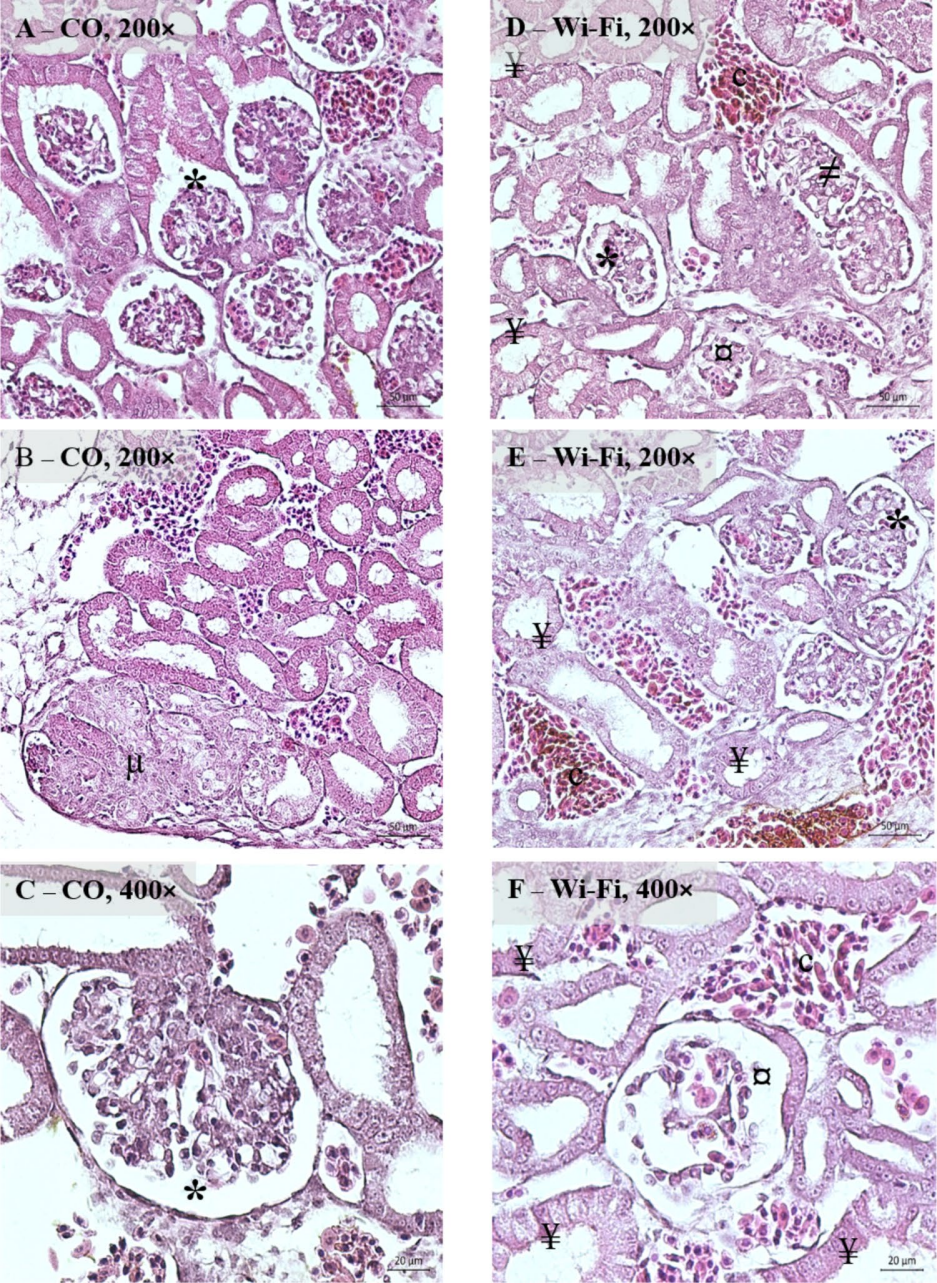
Representative microphotographs of the mesonephros of the 9-days old chicken embryo in the control and Wi-Fi groups **A** – **F**, *—renal corpuscles with typical structure, ≠—duplicated renal corpuscles, ¤—renal corpuscles with completely disintegrated structure, ¥—necrotizing cells, c – congested blood vessels, µ—focal physiological degeneration of mesonephric parenchyma (HE, magnification 200 × and 400 ×)

Despite the well-progressed organogenesis that was observed at the level of light microscopy in both investigated groups, several delicate structural changes in the Wi-Fi group were observed. Mesonephros in embryos from both groups (CO 1 and Wi-Fi 1) were covered by a thin connective tissue capsule and the developing parenchyma was composed of mesonephric corpuscles, mesonephric tubules and mesonephric duct. All above mentioned structural components were surrounded by a sparse interstitium with blood vessels of different calibre. In the control group, no signs of degeneration were present in most of the parenchyma, with the exception of focal physiological degeneration in the cranial part of the mesonephros (Fig. 4A − C). In the Wi-Fi group, the entire mesonephric parenchyma showed evident signs of diffuse degeneration. We focused on the cranial, middle and caudal regions of the mesonephros and the following structural changes were found: some mesonephric corpuscles had a relatively preserved structure with a well-defined glomerulus and capsule (Fig. 4D, E), but others showed complete structural disintegration (Fig. 4F). An increased incidence of duplicated renal corpuscles was found in the Wi-Fi group as well (Fig. 4D). The mesonephric tubules contained increased number of the shrunken, dead cells with dark nuclear chromatin and strongly eosinophilic cytoplasm. The interstitium in the Wi-Fi group contained markedly congested blood vessels, but no inflammatory infiltrations were present (Fig. 4D − F).

### TUNEL and MCM2 detection

**Fig. 5:**
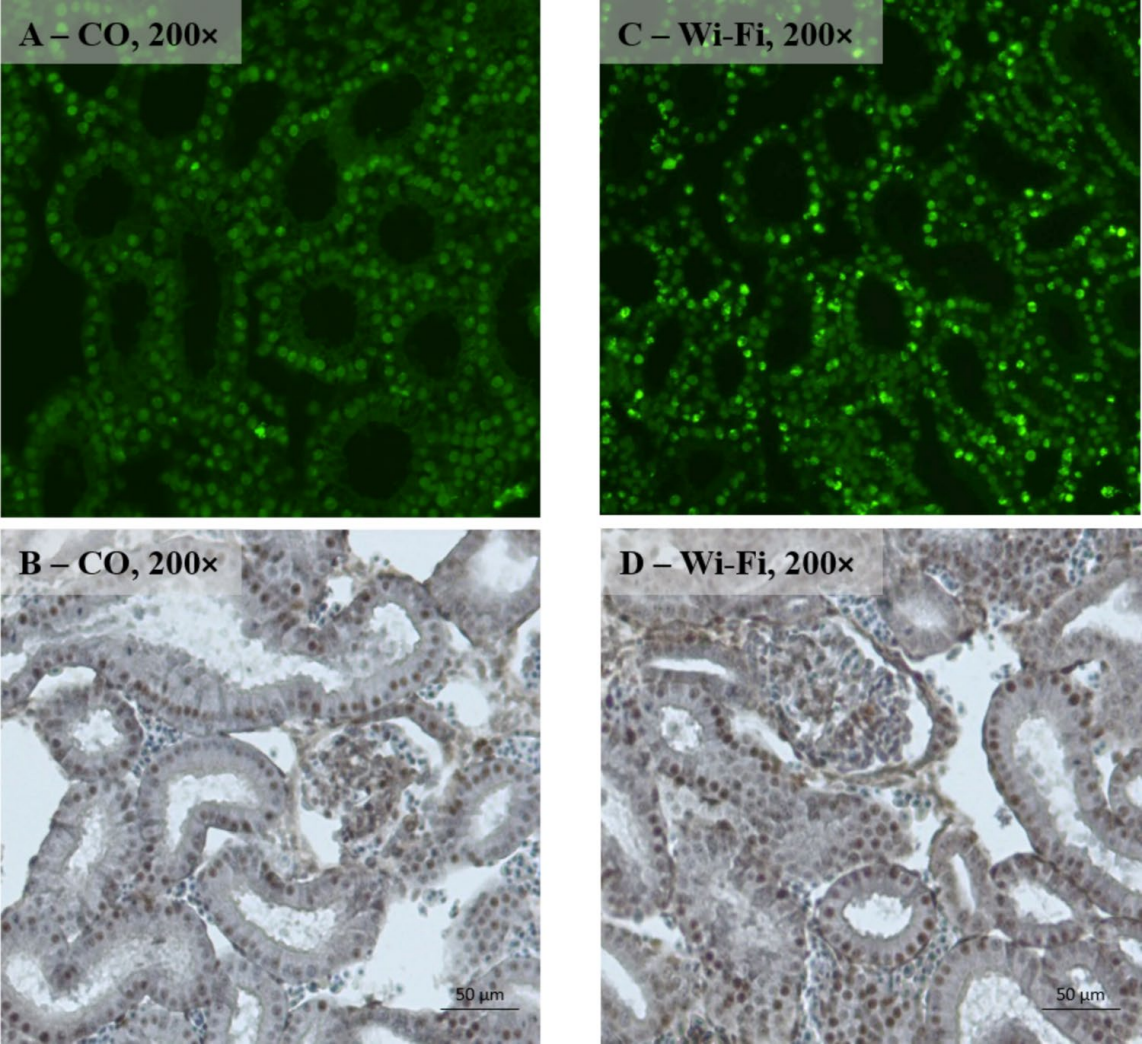
Representative microphotographs of mesonephros with TUNEL **A**, **C** and MCM2 **B**, **D** labelling (magnification 200 ×)

**Fig. 6:**
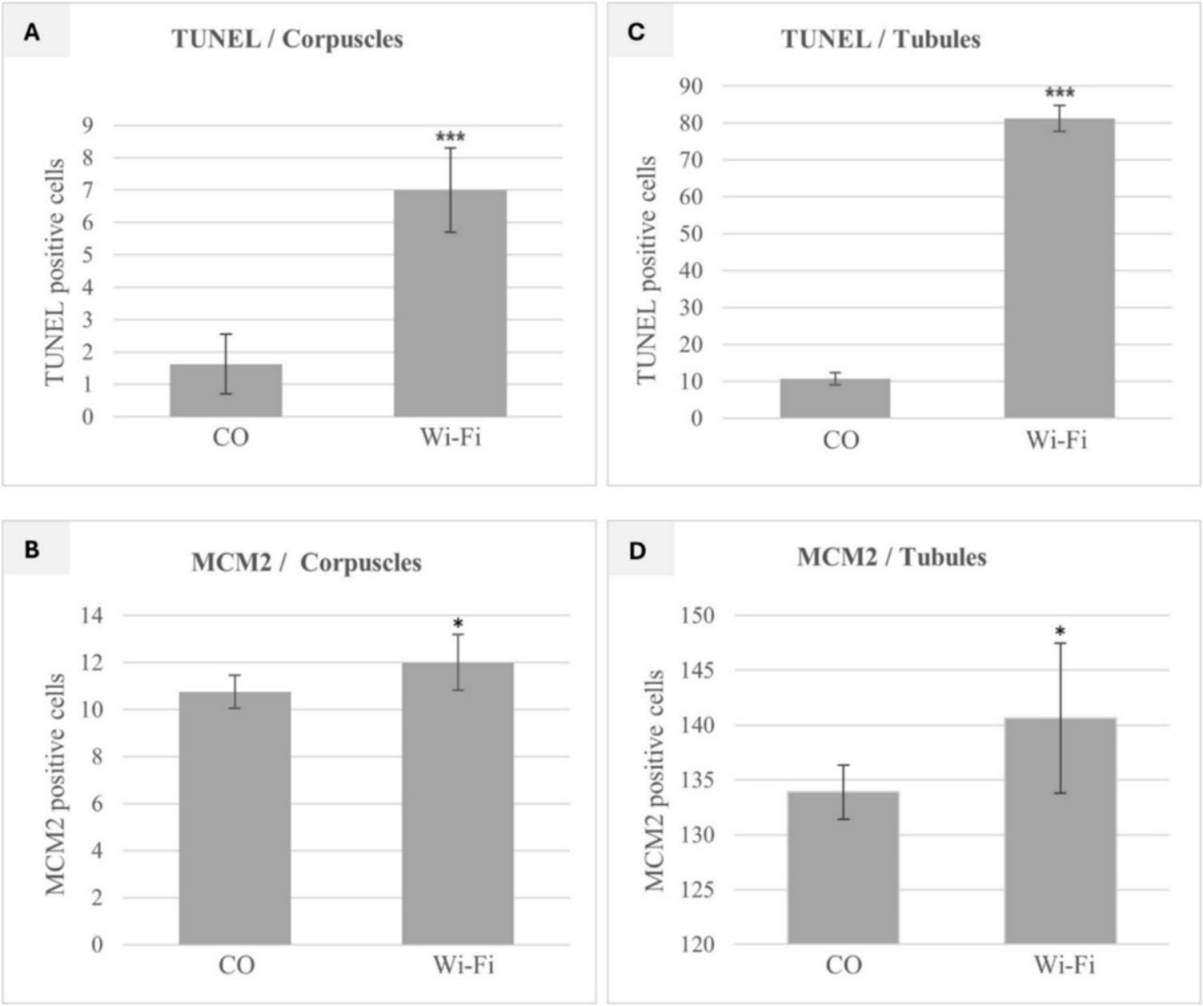
Number of TUNEL positive cells in mesonephric corpuscles **A** and mesonephric tubules **C**, with a significant increase (*p* < 0.001^***^) in the number of TUNEL positive cells in the Wi-Fi group regarding to control. MCM2 positive cells in mesonephric corpuscles **B** and mesonephric tubules **D**, with a significant increase (*p* < 0.05^*^) in the number of MCM2 positive cells in the Wi-Fi group compared to the control

TUNEL positive cells with an intense, bright green colour were present in the mesonephric tubules and mesonephric corpuscles in both control and Wi-Fi groups. However, mesonephric tubules and mesonephric corpuscles in the Wi-Fi group contained markedly higher number of TUNEL positive cells compared to the control (Fig. 5A, C). By a semi-quantitative evaluation we registered a significant increase (*p* < *0.001*) in the TUNEL positive cells in the Wi-Fi group compared to the control group (Fig. 6).

MCM2 positive cells, which showed an intense bright brown colouring, appeared in comparable numbers in tubules and corpuscles of both control and Wi-Fi groups (Fig. 5B, D). The semi-quantitative evaluation revealed a significant (*p* < *0.05*) increase in the number of MCM2 positive cells in the Wi-Fi group compared to the control group (Fig. 6).

### Relative gene expression for caspase – 1

**Fig. 7:**
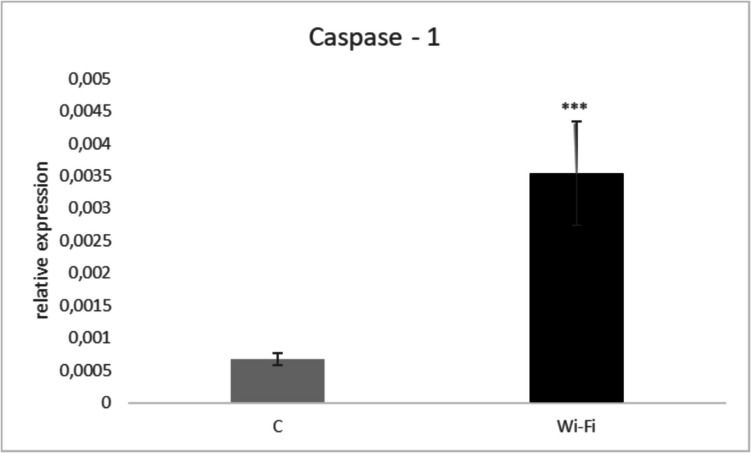
Relative gene expression for caspase- 1 in the chicken embryo mesonephros of the control and Wi-Fi group. Asterisk indicate significant differences among groups and time points at ***p < *0.001*

We recorded a significant upregulation (*p* < *0.001*) for caspase—1 in the Wi-Fi group in comparison to the control group (Fig. 7).

## Discussion

It is generally well known that NIR can affect living organisms in two possible ways. The first mode is the so-called thermal effect, caused by vibration and rotation of molecules in tissues with the subsequent temperature increase. The second one is a non-thermal or biological effect that induces oxidative stress, genetic damage, alteration of the cell membranes and many other changes at a cellular level (Hinrikus et al. 2005; Belyaev 2009; Pilla 2012; Park et al. 2014; Pall 2016; Ibitayo et al. 2017; Zhi et al. 2017; Das 2020; Roy et al. 2021). The type of the response of living systems to the NIR exposure varies and largely depends on the frequency, strength, distance and duration of the induced fields as well as position of the NIR source from the biological object. Space distribution of the fields within a biological systems and their further interactions with the tissues are markedly influenced by the character of the living matter. Common frequencies of the 2G, 3G, 4G, and 5G wireless standards are able to penetrate several centimetres deep into the tissues and become absorbed by them with subsequent release of energy. In general, lower frequencies of NIR penetrate deeper into the tissues (Belyaev 2009; Mild et al. 2019; Das 2020; Roy et al. 2021). Living body can generally adapt to a small and temporary temperature increase, but under a specific conditions, such as the small distance of the biological object from the radiation source, high water content of the tissue or presence of anatomical structures that prevent the efficient heat dissipation from the tissues as a result of which the thermoregulatory mechanisms of organism fail and general overheating or the so-called hot-spots may occur (Adair and Black 2003). Because the options for measuring temperature rise of the specific tissues caused by controlled exposure to experimental NIR are very limited, and in many cases completely impossible, determining whether the changes in tissues are due to the thermal or non-thermal effect of NIR is not attainable. In this study, we focused on evaluation, how the exposure to Wi-Fi with a frequency of 2.4 GHz at a low intensity of 200—500 µW/m^2^, which is very far below the permitted limits for both occupational (50 W/m^2^) and general public (10 W/m^2^) exposures (ICNIRP 2020; IEEE 2019), will affect the mesonephros development of the chicken embryo during a 9-day incubation period.

The kidney of birds, like that of mammals, develops in three subsequent stages. The first and middle stages, the pronephros and mesonephros degenerate, and the last stage, the metanephros transforms into the definitive organ. The avian mesonephros, although only a transitional structure, offers wide possibilities for studying various stem cells as well as the course of their differentiation. It becomes functional in birds from approximately the 5th to the 11th day of incubation and gradually begins to degenerate from its cranial toward the caudal end (Bellairs and Osmond 2014).

At the level of the conventional light microscopy, we noticed moderate, diffuse degenerative changes in the mesonephric parenchyma of the Wi-Fi group in addition to the physiologically occurring degeneration of the mesonephros in the both control and Wi-Fi groups. In these pathologically altered areas we noted complete structural disintegration of some mesonephric corpuscles, increased number of duplicated renal corpuscles as well as elevated number of necrotising cells. The surrounding interstitium contained many congested blood vessels. On the basis of the observed changes, especially the congestion of blood vessels, which can be considered as the activation of tissue thermoregulatory mechanisms, we assumed that in the tissue of the mesonephros, due to the influence of the wavelength at a specific power density of NIR used in the present 9-day experiment, the thermal effect occurred. The subsequent structural changes indicated moderately severe damage to the mesonephros, which pointed to the fact that even if the safety limits for the controlled exposure were not exceeded, the effects of NIR on organogenesis and the overall prenatal development of the birds should be considered. A strong argument is the fact that the entire embryonic development of a bird takes place in the egg, where individual layers such as the albumen and shell can prevent the effective removal of accumulated heat from the embryonic tissues. Similar phenomenon was observed in our previous experiment, where in the adult rats exposed to the NIR with a frequency of 2.45 GHz and power density of 28 W/m^2^ during 3 h a day for 3 weeks, congestion of testicular blood vessels occurred (Almášiová et al. 2017). Other authors noted this phenomenon especially in the organs which are particularly sensitive to the thermal effect due to their specific anatomy, such as high water amount, prominent fibrous capsule and surface placement in the organism, such the testes, eyes, salivary glands or brain (Deshmukh et al. 2015; Shahin et al. 2015; Dasdag and Akdag 2016; Zhi et al. 2017; Wainwright 2000).

Many scientific experiments have demonstrated changes at the cellular level attributed to the non-thermal effect of NIR at the power densities well below the permitted protective limits. These included DNA damage, oxidative stress, increased intracellular calcium, increased expression of stress proteins, enhanced apoptosis, and changes in cell proliferation (Hinrikus et al. 2005; Belyaev 2009; Deshmukh et al. 2015; Shahin et al. 2015; Dasdag and Akdag 2016; Othman et al. 2018; Das 2020). Especially, prenatal development is a critical period of life, characterized by extreme sensitivity of developing tissues and organs to various environmental risk factors, including NIR (Lee et al. 2014; Zhang et al. 2016; Sage and Burgio 2017; Othman et al. 2018) which must not go unnoticed and deserves the proper attention.

In agreement with the above findings, we also observed statistically significant changes in proliferation as well as apoptosis of embryonic cells in the current experiment. The number of apoptotic cells was semi-quantitatively evaluated via sophisticated TUNEL assay that detects DNA strand breaks in the tissue sections. We noticed that in the Wi-Fi group a significant increase (p < 0.001) was detected in the rate of apoptosis compared to the control group. It is generally known that apoptosis plays an important role in many physiological conditions, including normal tissue development and organogenesis and balancing the mitotic division. However, the excessive cell death that was induced due to NIR, can finally lead to a harmful cell loss with the subsequent disturbances of the normal structure as well as functioning of the organ. On the other hand, especially initial developmental stages are characterized by the intense proliferation of embryonic cells, which then differentiate to form specialized cell types. As cells progressively differentiate, their rate of proliferation usually decreases, and most cells in adults are arrested in the G0 stage of the cell cycle. In the present experiment we used the MCM2 (Minichromosome Maintenance Protein) as an important participant in DNA replication to demonstrate proliferating cells in the mesonephros parenchyma. The semi-quantitative evaluation of the MCM2 positive cells within the mesonephric tubules and corpuscles revealed their significant (p < 0.05) increase in the Wi-Fi group in comparison to the control group which is consistent with the findings of Lee et al. (2014), who observed higher proliferation rate due to Wi-Fi signal in their in vitro study carried out on adipose-derived stem cells.

To our knowledge, this is the first experimental study that investigated the effects of Wi-Fi on the histological structure and other selected parameters in chicken mesonephros. A novel approach consisted also of the focus on monitoring the effect of Wi-Fi radiation on the gene expression of caspase-1. Generally, caspases are cysteine ​​proteases that act either in the process of apoptosis or during inflammatory responses, such as caspase- 1. Caspase- 1 is a part of the cytosolic multiprotein inflammasome complex (NALP3) which represents the first line of defence during cellular stress and is one of the important components of innate immunity (Molla et al. 2020; Sahoo et al. 2024). In agreement with the previous statement, our results showed a negative effect of exposure to Wi-Fi radiation which was reflected in the up-regulation of gene expression for caspase- 1 in the embryonic mesonephros, which may indicate the occurrence of cellular stress and triggering potential inflammatory response. In addition, the embryonic cells are extremely sensitive to oxidative stress due to intensive cell division and the concomitant exposure of their DNA (Aouache et al. 2018). Our results may indicate that Wi-Fi radiation is involved in the development of an undesirable inflammatory response. Similarly, Vafaei et al. (2020) found that Wi-Fi radiation increases oxidative stress as well as the degree of apoptosis in placental tissue in mice. On the other hand, the majority of authors focused mainly on monitoring of the impact of Wi-Fi radiation on the activity of caspase- 3 or caspase- 8 and 9 in relation to the apoptosis in embryos (Cig and Naziroglu 2015; Saygin et al. 2018; Wlodarczyk and Nowicka 2019). Therefore, we assume that our results may also expand the current knowledge on the effects of Wi-Fi radiation on the gene expression for caspase-1 in the chick embryo. However, it was confirmed that increased gene expression for caspase-1 was observed not only in the amniotic fluid of pregnant women (Maneta et al. 2015) but also in chicken embryos on days 12 and 15 of embryogenesis (Grzegorzewska 2012).

In our study, we investigated the effect of Wi-Fi radiation on the mesonephros organogenesis in the chicken embryo mainly from the morphological aspect. We also evaluated the level of cell necrotising, proliferation, and relative gene expression for caspase- 1 within the observed groups. Permanent Wi-Fi exposure at the frequency of 2.4 GHz and power density of 200—500 µW/m^2^ from the first to the ninth day of the embryo incubation caused moderate, diffuse degenerative changes as well as enhancement of proliferation, apoptosis and relative gene expression for caspase-1 in the mesonephros parenchyma. All the observed changes clearly indicated damage to developing kidney tissue and proved the triggering of the potential inflammatory tissue response. Blood vessels congestion in the interstitium also supported the assumption that a thermal effect was induced in the mesonephros tissue even though the safety limits for the NIR were not exceeded. Although the results of our study cannot be directly extrapolated to humans, the results observed represent a potential health hazard and indicate the need to observe the so-called precautionary principles and consider the risk of excessive exposure to NIR sources with respect to children, adolescents and especially during pregnancy.

## Data Availability

No datasets were generated or analysed during the current study.
